# Genetic Diversity of the Non-Polio Enteroviruses Detected in Samples of Patients with Aseptic Meningitis in the Ural Federal District and Western Siberia

**DOI:** 10.3390/v18010121

**Published:** 2026-01-16

**Authors:** Tarek M. Itani, Vladislav I. Chalapa, Anastasia K. Patrusheva, Evgeniy S. Kuznetsov, Aleksandr V. Semenov

**Affiliations:** 1 Federal Budgetary Institution of Science, «Federal Scientific Research Institute of Viral Infections «Virome»», Federal Service for Surveillance on Consumer Rights Protection and Human Wellbeing, Ekaterinburg 620030, Russia; chalapa_vi@niivirom.ru (V.I.C.); patrusheva_ak@niivirom.ru (A.K.P.); kuznecov_es@niivirom.ru (E.S.K.); semenov_av@niivirom.ru (A.V.S.); 2 Department of Medical Microbiology and Clinical Laboratory Diagnostics, Ural State Medical University, Ekaterinburg 620109, Russia; 3 Institute of Natural Sciences and Mathematics, Ural Federal University Named After the First President of Russia B.N. Yeltsin, Ekaterinburg 620075, Russia

**Keywords:** aseptic meningitis, NPEV, genotypic composition, next-generation sequencing (NGS), E30, E6, CVA9, phylogenetic analysis

## Abstract

Human non-polio enteroviruses (NPEVs) cause a plethora of infections in humans, ranging from mild to severe neurological diseases including aseptic meningitis. NPEVs are the leading cause of aseptic meningitis in both children and adults worldwide. In Russia, reports of NPEV infections have surged, especially in the post-COVID era starting in 2022, with elevated infection rates into 2023. A comprehensive examination of the whole genome is crucial for understanding the evolution of NPEV genes and for predicting potential outbreaks. This study focused on identifying the circulating NPEV strains in the Ural Federal District and Western Siberia, using Sanger sequencing and next-generation sequencing (NGS) methodologies. Biological samples were collected from (*n* = 225) patients diagnosed with aseptic meningitis. Bioinformatics analysis targeted the nucleotide sequences of the major capsid protein (partial VP1) gene fragment, and the assembly of whole NPEV genomes. A total of 159 NPEVs were characterized, representing 70.7% of the collected samples. The main capsid variants forming the predominant genotypic profile included E30 (*n* = 39, 24.3%), E6 (*n* = 31, 19.3%), and CVA9 (*n* = 25, 15.6%). Using NGS, we successfully assembled 13 whole genomes for E6, E30, EV-B80, CVA9, CVB5, E11, and EV-A71 and 3 partial genomes for E6 and EV-B87. This molecular-genetic analysis provides contemporary insights into the genotypic composition, circulation patterns, and evolutionary dynamics of the dominant NPEV associated with aseptic meningitis in the Ural Federal District and Western Siberia. The laboratory-based monitoring and epidemiological surveillance for genetic changes and evolutionary studies are important for improving prevention and healthcare.

## 1. Introduction

Non-polio enteroviruses (NPEVs) constitute the largest genus within the Picornaviridae family. These are small naked single-stranded positive RNA viruses of approximately 7500 nucleotides grouped into four species, *Enterovirus alphacoxsackie* (EV-A), *Enterovirus betacoxsackie* (EV-B), *Enterovirus coxsackiepol* (EV-C), and *Enterovirus deconjuncti* (EV-D), and more than 100 distinct genotypes [1,2]. Most viruses in this genus cause infections in humans, ranging from mild respiratory illnesses to severe neurological diseases [3]. Clinical manifestations may develop after an incubation period of 3 to 21 days. NPEVs are distributed worldwide and have a seasonal incidence pattern in temperate regions during summer and fall, while they occur year-round in tropical regions [4].

NPEVs are associated with a range of central nervous system infections and are the leading cause of aseptic meningitis in adults and children worldwide [5,6]. Aseptic meningitis is a clinical entity characterized by an elevated leukocyte count in the cerebrospinal fluid (CSF), typically manifesting as pleocytosis. This condition is distinguished by the absence of positive findings on Gram stain and bacterial culture. Furthermore, aseptic meningitis is defined by the lack of a discernible parameningeal focus or systemic illness, underscoring its etiological heterogeneity and the challenges in identifying its causative agents. This leads to favorable clinical outcomes [7] and manifests differently depending on the patient’s age and immune health [8]. Nearly 50 NPEV types have been identified as causes of aseptic meningitis globally, distributed across the four species [9]. EV-B types including CV-B3-5, CVA9, and E6, 9, and 30 are the most identified in patients with aseptic meningitis [5] with E30 as the most common cause of viral meningitis and reported in outbreaks worldwide [8,10].

The NPEV genome includes a long single open reading frame (ORF) flanked by 5’UTR and 3’UTR. The ORF encodes a single polyprotein that can be cleaved into mature viral capsid proteins P1 (VP4, VP2, VP3, and the major capsid protein VP1) and non-structural proteins P2 (2A–C) and P3 (3A–D). VP1, VP2, and VP3 are on the surface of the viral capsid and are under immune pressure, whereas VP4 is inside the capsid [11]. VP1 is the most external and immunodominant capsid protein that contains the neutralization epitopes, and can be used for NPEV genotyping [12]. Currently, Sanger sequencing of the VP1 capsid protein gene is the gold standard for NPEV genotyping. But whole-genome sequencing has become increasingly affordable, accessible, and cost-effective, which will likely allow improved typing in the near-future [13].

There is limited information on circulating NPEV genotypes in aseptic meningitis patients from the Ural Federal District (UFD) and Western Siberia (WS). Such knowledge is essential for laboratory diagnostics, patient management, and future outbreak responses. To gain a deeper understanding of the epidemiology of aseptic meningitis for the period spanning 2022 to 2024, this study aimed to identify the NPEV circulating in the Ural Federal District and Western Siberia population using VP1 gene typing and whole-genome sequencing (WGS). Additionally, this research aimed to investigate the correlation between NPEV types and the genomic evolution of the disease.

## 2. Materials and Methods

### 2.1. Ethical Consideration

This research was performed as part of the Russian state program for NPEV surveillance. Since 2017, national enterovirus surveillance has been collecting laboratory data and biological samples from enteroviral infections in the UFD and WS. The participating centers were asked to report NPEV meningitis/encephalitis detections monthly to the Ural-Siberian regional scientific and methodological center for the study of enteroviral infections. According to national regulations, the use of anonymous samples and data from state epidemiological surveillance does not require informed consent. The research was performed with a waiver of consent under IRB № 4 of the Local Ethics Committee of the State Scientific Center of Virology and Biotechnology “Vector” (date of approval 24 June 2022).

### 2.2. Clinical Samples and Data Collection

A total of 281 positive samples from 225 patients with NPEV meningitis were received in the Laboratory of Enteric Infections from January 2022 to the end of December 2024. These samples included 119 residual CSF samples positive for NPEV from patients with confirmed aseptic meningitis, which were collected by participating centers, analyzed by real-time PCR amplification using “AmpliSens Enterovirus-FL” (InterLabService Ltd., Moscow, Russia), and transported to our laboratory at approximately 4 °C. Additionally, pharyngeal swabs (*n* = 89) and stool specimens (*n* = 73) were included in our study. Pharyngeal swabs (*n* = 58) and stool samples (*n* = 48) were also included in our study when CSF samples were not available or untypable (Table 1). All samples were stored at −20 °C until processing. For laboratory studies, stool suspensions were prepared by adding approximately 0.1–0.2 g of each stool sample to polypropylene test tubes, containing 1–1.5 mL of PBS.

### 2.3. Enterovirus Diagnosis and Molecular Genotyping

First, the samples were centrifuged for 2 min at 10,000× *g* at room temperature in order to remove the solid particles. Viral RNA genome was isolated from CSF, pharyngeal swabs, and stool suspensions employing the RIBO-prep kit (InterLabService Ltd., Moscow, Russia) according to manufacturer’s protocol. cDNA synthesis was carried out using the REVERTA-L-100 kit (InterLabService Ltd., Moscow, Russia). Next, two rounds of PCR targeting the VP1 gene were performed according to Nix et al. with minor modifications as previously described [14]. The reaction products were separated and visualized on 1.5% agarose gels and extracted by using the Cleanup Standard reagent kit (Evrogen JSC, Moscow, Russia). Purified products were sequenced in both directions (forward and reverse) using the deoxy sequencing Sanger method with the BigDye™ Terminator v3.1 Cycle Sequencing Kit (Applied Biosystems, Austin, TX, USA) on an automatic genetic analyzer 3130 Genetic Analyzer (Thermo Fisher Scientific, Waltham, MA, USA), following the manufacturers’ protocol.

### 2.4. Virus Isolation, Preparation of Libraries, Whole-Genome Sequencing

**Figure 1:**
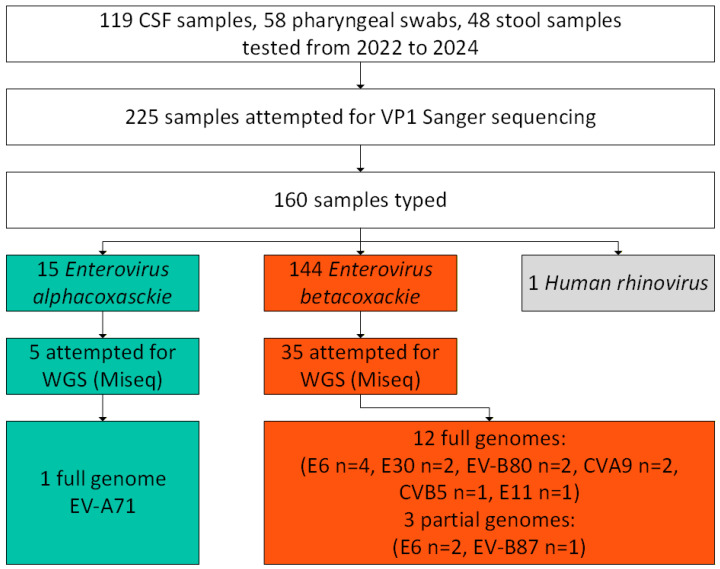
Flow diagram of the study cohort and sequenced samples in aseptic meningitis patients from in the Ural Federal District and Western Siberia. *Enterovirus alphacoxackie* are shown in cyan. *Enterovirus betacoxackie* are shown in orange.

All the samples that were attempted for WGS were inoculated on RD (Rhabdomyosarcoma) for the detection of non-polio enteroviruses as per the WHO non-polio enteroviruses Guidelines (Figure 1). Briefly, a volume of 0.2 mL of treated fecal sample, CSF, or pharyngeal swab suspension was used for the RD inoculation. The inoculated tubes were then incubated at 37 °C for a period of 1–10 days, until a complete cytopathic effect was observed under an ordinary light microscope. Uninfected cells were used as a negative control. NPEV strains isolated on RD cell lines were directed to WGS. Cells were frozen and thawed three times and then subjected to an RNA extraction procedure. A volume of 200 µL of the cell culture supernatant was used for total RNA extraction with ExtractRNA reagent (Evrogen JSC, Moscow, Russia) for further molecular biological analysis. The RNA concentration was determined on a Qubit 4.0 fluorimeter using the Qubit RNA Assay kit (Qubit™ RNA BR Assay, Thermo Fisher Scientific, Waltham, MA, USA). To confirm the isolation of NPEV RNA, real-time PCR amplification was performed using a set of reagents “AmpliSens Enterovirus-FL” (InterLabService Ltd., Moscow, Russia) in a StepOnePlus amplifier (Applied Biosystems, Austin, TX, USA).

cDNA synthesis was performed using the Mint cDNA synthesis kit (Evrogen JSC, Moscow, Russia). DNA libraries were obtained with the NEB Next Ultra II RNA Library Prep Kit (New England Biolabs, Ipswich, MA, USA) and DNA concentration was measured after library preparation on a Qubit 4.0 fluorimeter using the Qubit dsDNA HS Assay Kit (Thermo Fisher Scientific, Waltham, MA, USA) for the quantitative determination of double-stranded DNA. The quality of each library was assessed by analyzing a volume of 2 µL using a Qsep One Plus fragment analyzer (BioOptic, New Taipei City, Taiwan) with a high-resolution cartridge and by visualizing on a 2% agarose gel (TBE buffer, 180 V for 30 min). Prepared libraries were pooled into a single tube pool of 4 nM by dilution in sterile water. The concentration of the final library pool was measured using the QubitTM dsDNA HS Assay Kit. Genomic libraries were sequenced on the Illumina MiSeq platform (Illumina, San Diego, CA, USA), utilizing the MiSeq v2 cartridge, which generates paired readings of 2 × 250 cycles. All kits were used following the manufacturer’s instructions.

### 2.5. Bioinformatic Analysis for Sanger and WGS

All VP1 sequences were visualized using Chromas 2.6.6 (Technelysium, Pty, Ltd, South Brisbane, QLD, Australia. version 2.6.6) and NPEV genotyping was carried out by comparing the results of direct genome sequencing of NPEV with the reference sequences presented in the international GenBank database of genetic data and the BLAST service (https://blast.ncbi.nih.gov/, accessed on 19 June 2025). In addition, genotyping was confirmed using the online RIVM program (https://www.rivm.nl/mpf/typingtool/enterovirus/, accessed on 2 July 2025).

For WGS, low-quality reads were filtered, and adaptors were trimmed with Trimmomatic. The quality of the read was visualized using FastQC software V.0.11.9 before and after trimming. De novo assembly was performed using SPAdes genome assembler V3.9 [15] programs with default parameters.

### 2.6. Phylogenetic and Statistical Analysis

Using MEGA software V11 [16], nucleotide and amino acid sequences were aligned and subjected to preliminary analysis through ClustalW procedures. Next, representative whole-genome sequences—one for each identified genotype—were added to the alignments from the GenBank database. Then, alignments were analyzed in BEAST software V1.10.4 [17] while parsing collection years for each strain and performing calibration of the strict molecular clock for EV-A71 because of the authors’ concern about its origin (a strict clock was used to prevent over-parameterization). The evolutionary models for all trees were GTR+G+I based on the results of ModelTest-NG software V0.1.7 [18]. The obtained phylogenetic trees were inspected using Tracer software V1.7.2 [19], finalized in TreeAnnotator software V1.10.4 (part of BEAST package) and visualized in FigTree software V1.4.4 [20] with minimal graphical adaptations. During the procedure, no formal model selection was conducted.

## 3. Results

### 3.1. Incidence of Enterovirus Meningitis in the Ural Federal District and Western Siberia

**Figure 2:**
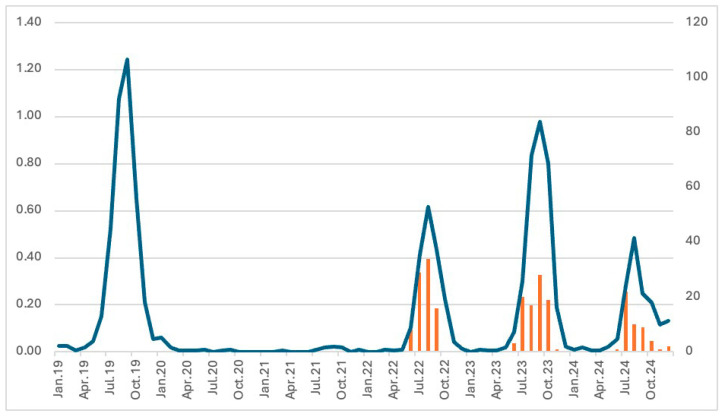
Long-term dynamics of the incidence rates of NPEV aseptic meningitis in UFD and WS during 2019–2024, and monthly incidence rates per 100,000 inhabitants (curve in blue) and number of collected samples from 2022 to 2024 (histograms in orange).

We analyzed the data on aseptic meningitis that was provided to the Ural-Siberian regional scientific and methodological center for the study of enteroviral infections from January 2019 to December 2024. All these cases were laboratory-confirmed, but not fully genotyped by partial VP1-sequencing. This allowed us to better understand the incidence of enterovirus meningitis in our region, and to better understand the pre- and post-pandemic context. Overall, 2465 cases of NPEV-associated aseptic meningitis were recorded in the UFD and WS (Figure 2). The average monthly incidence rate was 0.19 per 100,000 inhabitants with most cases occurring in the Sverdlovsk oblast (40%; 987/2465), Khanty-Mansi autonomous okrug (13.9%; 342/2465), and Novosibirsk oblast (12%, 295/2465). A notable increase in the number of aseptic meningitis NPEV-positive cases was observed in September–October 2023, declining in November 2023 and peaking again in the summer of 2024. The monthly incidence showed a summer-autumn pattern with 94.85% of the cases (2338/2465) occurring from June until November, with a peak in August–September. The long-term dynamics of the incidence of NPEV aseptic meningitis were characterized by a large decrease in 2020 and an increase in 2022, with a return to pre-pandemic values in 2023.

The most common clinical symptoms were fever, headache, and vomiting. A total of 281 positive samples from 225 patients with NPEV meningitis were received in the Laboratory of Enteric Infections from January 2022 to the end of December 2024. We analyzed and genotyped 225 samples (119 CSF, 58 pharyngeal, and 48 fecal samples). The age of the patients ranged from 1 year to 57 years, with a median of 8 years, and the male-to-female ratio was 1:0.63 (138 males/87 females). Schoolchildren, from 7 to 17 years, constituted 57.3% of the patients (129/225). Preschool children, from 3 to 6 years, constituted the second largest age group at 27.1% (61/225).

### 3.2. Partial VP1 Genotyping

**Figure 3:**
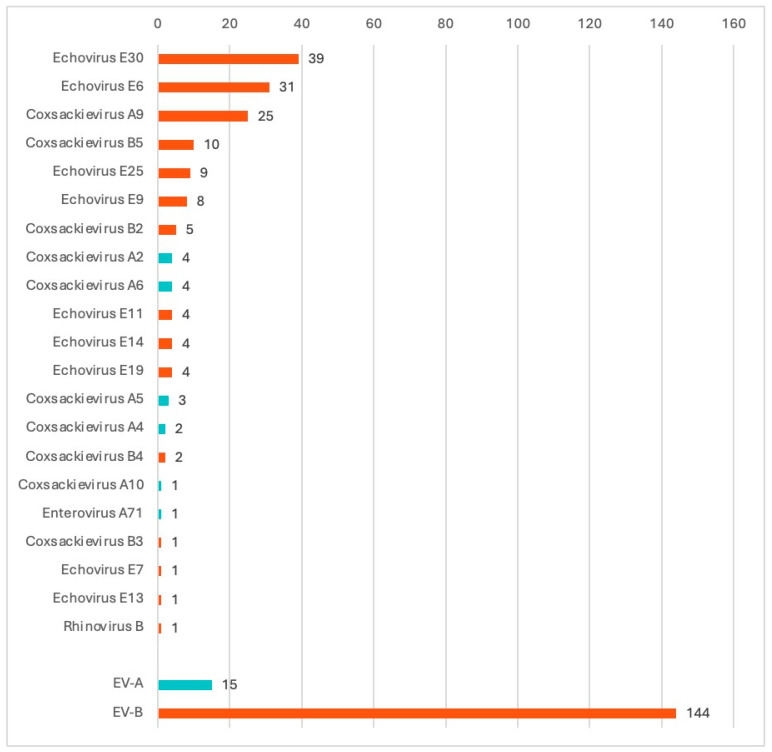
Species and types of NPEV isolated in aseptic meningitis in the Ural Federal District and Western Siberia (*n* = number of cases). *Enterovirus alphacoxsackie* are displayed in cyan histograms. *Enterovirus betacoxsackie* are displayed in orange histograms.

From the 225 aseptic meningitis patients, NPEV genotyping was successfully performed in 159 (70.7%), and more specifically 84/119 CSF samples (70.6%), 42/48 stool samples (87.5%), and 33/58 pharyngeal swabs (56.9%), indicating that stool and CSF samples are the most suitable biological samples for meningitis diagnosis. One nasopharyngeal sample typed as rhinovirus B, and was excluded from the analysis. When multiple samples from the same patient were analyzed, we detected the same NPEV genotype in all tested samples. VP1 genotyping revealed twenty-one different NPEV genotypes circulating among patients with aseptic meningitis in the UFD and WS (Figure 2). Most genotypes belonged to the *Enterovirus betacoxsackie* (EV-B) genus (90%), and less to the *Enterovirus alphacosackie* (EV-A) genus (9.4%), and only one rhinovirus was detected (0.6%, belonging to a different genus). Some Federal districts reported a low number of registered cases and sent a few samples. Most samples were received and typed from the Sverdlovsk oblast, Khanty-Mansi autonomous okrug, and Novosibirsk oblast (Table 2). Only two samples were successfully typed in the Omsk oblast (from two samples sent to our center) and one case was typed in the Tyumen oblast (from three samples sent). Three Federal districts did not send samples from aseptic meningitis patients (Yamalo-Nenets autonomous okrug, Kemerovo oblast, and Altai krai). The most prevalent genotypes were E30 (24.3%, 39/160), E6 (19.3%, 31/160), CVA9 (15.6%, 25/160), CVB5 (6.25%,10/160), and E25 (5.6%, 9/160) (Table 2 and Figure 3). EV-A species were mainly detected in children less than 6 years (12 out of 15 detected) and from the Sverdlovsk oblast (14 out of 15 detected). E30 was mainly detected in the schoolchildren group from 7 to 17 years (32 out of the 39 detected E30), while E6 was almost exclusively detected in patients from 3 to 18 years (28 out of the 31 detected E6). The sole EV-A71 strain was isolated in a patient with aseptic meningitis from a nasopharyngeal sample.

### 3.3. Whole-Genome Sequencing (WGS) and Sequence Assembly

Forty NPEV strains (35 EV-B and 5 EV-A) from respective clinical cases were isolated initially by cell culture on VP1 (RD cells). We chose the most predominant NPEV types found by VP1 genotyping: E30, E6, CVA9, CVB5, CVA2, and the potentially neurovirulent EV-A71. Due to the reduced sensitivity that is frequently encountered with conventional cell culture systems, repeated passages were required in most cases until the observation of a cytopathic effect. WGS was attempted with all these NPEV isolates using an Illumina MiSeq and only 16 were successfully sequenced by the NGS method. There were differences between the subsequent culture/NGS typing and the VP1 typing for three samples. Two samples were typed as E6 and one sample as E30 (1 sample) by the VP1 approach, while they were typed by the NGS method as EV-B80 (2 strains) and EV-B87 (1 strain). A list of the sequenced genome is provided in Table 3.

### 3.4. Phylogenetic Analysis of Full-Length Sequences

**Figure 4:**
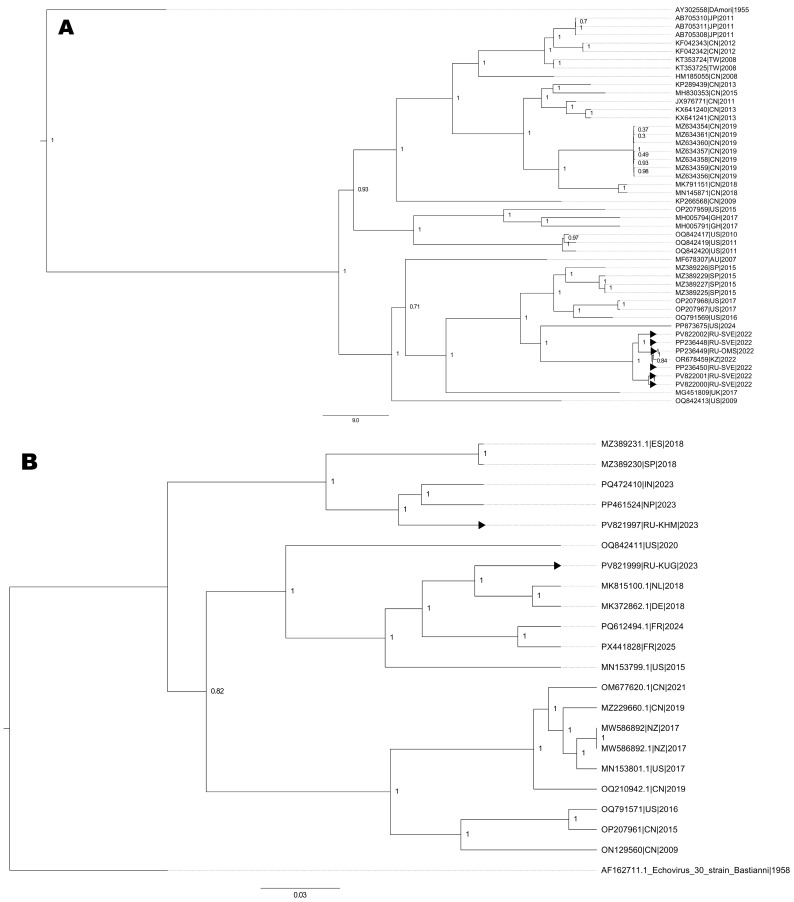

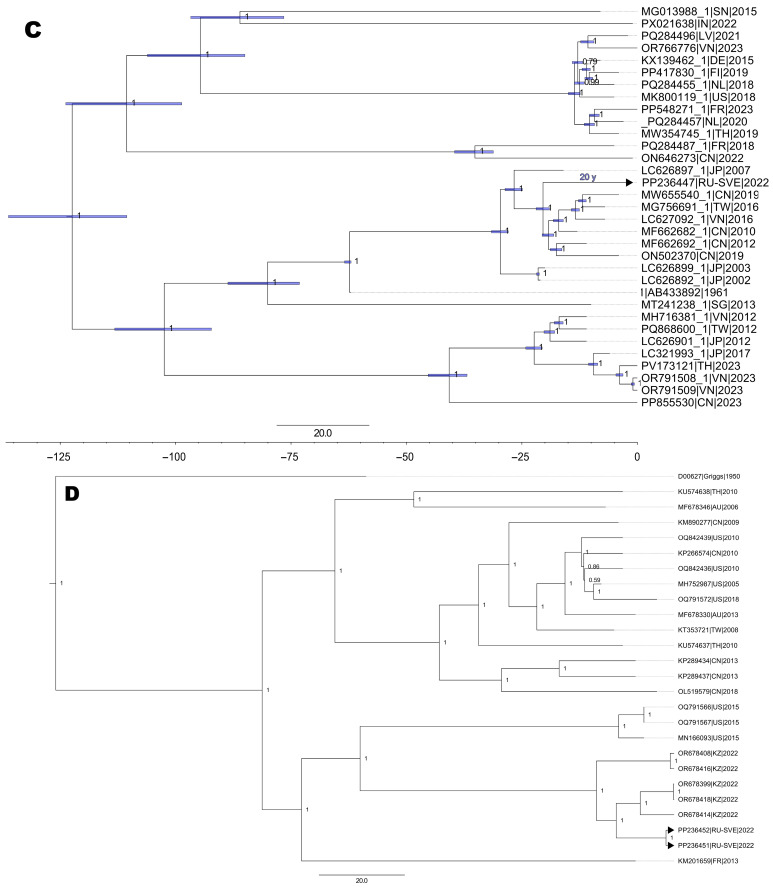
Cladogram of nucleotide sequences from full-length genomes: (**A**) six E6 strains, (**B**) three E30 strains, (**C**) one EV-A71 strain (blue bars correspond to Time to Most Recent Common Ancestor, tMRCA (in years), depicted above the branch), and (**D**) two CVA9 strains. Black arrows indicate full-length genome sequences of NPEV strains identified in the Ural Federal District and Western Siberia. Numbers at each branch correspond to its posterior probability values. For (**A**,**B**,**D**), the scale represents genetic distance, and (**C**) is scaled by time.

Phylogenetic analysis of E6 genovariants (six isolates) showed that most of the identified enteroviruses circulated in the studied regions for some years. As shown in Figure 4A, strains of E6 clustered closely with strains circulating in Baltimore, USA, from 2016 to 2017 [5] and strains that circulated in Spain during 2015 [13], while they were distant from Chinese E9 strains reported between 2013 and 2019.

Concerning the reconstruction of phylogenetic events for nucleotide sequences from two E30 full-length genomes, the topology of the cladogram demonstrates the formation of two distinct nodes (Figure 4B): (i) PV821997.1 clustered with MZ389231, an E30 strain circulating in Spain in 2018 [13], forming a cluster (sub-genogroup II), but was still 10% different; (ii) another cluster consisted of PV821999.1 that formed a monophyletic cluster with strains from Germany and the Netherlands in 2018 (sub-genogroup V).

Phylogenetic reconstruction, involving nucleotide sequences from full-length genomes CVA9 circulating in the Sverdlovsk oblast, show the formation of an internal clade with a hypothetical common ancestor among CVA9 (Figure 4C). These CVA9 strains formed a common cluster with sequences from Kazakhstan, without polyphyletic relationships with sequences from China.

Finally, EV-A71 strains were clustered with strains from the Asian-Pacific region (Figure 4D), but the estimated age of the most recent common ancestor (Time to Most Recent Common Ancestor, tMRCA) was 20 years (95% HPD 18–21) and belonged to subgroup C4.

## 4. Discussion

Aseptic meningitis, the most frequent infection of the central nervous system, is caused by viruses, bacteria, fungi, and parasites, with viruses being the predominant agents [21]. NPEVs are the primary viral agents responsible for this condition. Hence, rapid NPEV identification and genotyping are critical for patients with aseptic meningitis. NPEV meningitis outbreaks have been frequently reported in the Russian Federation [22].

There is little current data on NPEV circulating in the UFD and WS population with aseptic meningitis. This study describes the NPEV types detected in samples from aseptic meningitis patients (CSF, Feces, NP), as part of the National Surveillance Program from January 2022 to December 2024. A total of 281 NPEV-positive samples were received, and partial VP1 gene sequencing was attempted with 225 samples and WGS was attempted with 40 samples.

The monthly incidence shows a summer-autumn pattern, which is typically observed in temperate regions. The COVID-19 pandemic halted the spread of NPEV, likely due to stringent infection control measures [5]. As these measures relaxed, the circulation and incidence of NPEV meningitis surged in our region. In our cohort, most patients with positive NPEV were schoolchildren (7 and 17 years). Over the three-year study period, partial VP1 genotyping showed that EV-B species E30, E6, and CVA9 were the major identified types in aseptic meningitis patients. Minor genotypes recorded were CVB5, E25, and E9. EV-B species have been globally the most frequent in aseptic meningitis in Europe [23,24,25,26], in Africa [6,27,28,29], in Asia [2,8,12,30,31], in the Middle East [32,33], and in the US [5].

E30 was mainly detected in schoolchildren, which is consistent with a British study by Holmes et al. [23] but differs from previously published reports where E30 was mainly identified in adults [5,27]. E6 was the predominant type in 2024, which is in alignment with previous studies where E6 was the predominant type before the COVID-19 pandemic in 2018 and 2019 [8,30]. Also, CVA9 was reported as a major type in aseptic meningitis cases worldwide including the USA [5]. In addition, the EV-A71 strain subgenogroup C4, mainly circulating in Asia, was identified in this study. This strain is considered highly neurovirulent and accounts for 80% of severe and 93% of fatal HFMD cases in China [34]. The EV-A71 subgroup C4 was reported in several European countries including France [35] and the city of Rostov-on-Don in Russia [22]. Nevertheless, the identified EV-A71 strain has a large evolutionary distance from tMRCA. The authors interpret this result with caution because of possible selection bias during the building of alignment (only whole-genome sequences were included with its linkage to meningitis cases).

Because CSF contains low enteroviral copies, it is recommended to test and type additional respiratory and stool samples [36]. In this study, VP1 genotyping was higher in stool samples (87.5%) compared to CSF (70.6%) and pharyngeal samples (56.9%). In all cases, the NPEV genotype was identical in all tested samples, when applied. This result is in accordance with a Greek study [26]. This finding is important for clinicians as CSF quantity is usually limited for molecular analysis especially in neonates and infants.

Schoolchildren accounted for the highest number of NPEV-positive cases, making up about 60% of all infections. This finding contrasts with a study from Palestine [32], which reported the highest rates among infants under one year old.

The circulation of multiple NPEV genovariants may be affected by various factors such as viral evolution, recombination events, population bottlenecks, virulence, host immunity, and the introduction of novel types [37].

The success rate of direct NPEV typing was high (70.6%), which is significantly higher than in previous studies at 21.2% [8] and 50% [27]. The high detection rate of NPEV infections might be attributed to effective reporting practices, careful sample transportation, and the refined PCR conditions in the Nix nested-PCR protocol [38].

There are some limitations to this study: first, the number of samples is relatively small; second, the epidemic circulation patterns of different types of NPEV, with years of high incidence (e.g., E30), are followed by others with low or very low detection; third, the NGS protocol required the growth of NPEV on cell culture, which is a limiting factor as many aseptic meningitis viruses are characterized by poor growth (EV-A71) or no growth of CVA1-A6 [28].

Limited data are available regarding circulating genotypes, which are associated with aseptic meningitis in Russia and in Europe during this period [39]. E30 was the predominant NPEV genotype in our area in 2023. E30 meningitis outbreaks have been reported to occur worldwide every 3 to 5 years [12,38]. Recent E30 meningitis outbreaks occurred in Germany [25] and in Northern Europe [40].

Other similar studies reported totally different results with a predilection for the infant and adult age group [23,41]. This may be due to variation in the health-seeking behavior between different populations. Outdoor activity is probably the main reason why the male population was more infected by NPEV than the female population, with more aseptic meningitis cases in males. These results are consistent with previous reports [12].

In this study, a preliminary phylogenetic analysis of E30, CVA9, and E6 strains was performed and indicated a possible correlation with other strains circulating in Spain (E6, E30), Germany and the Netherlands (E30), the USA (E6), and Kazakhstan (CVA9), which could be attributed to migration and population movement.

A large proportion of the NPEV-positive samples originated from preschool/schoolchildren from 3 to 17 years (190 out of the 225 included patients). An interesting observation of this data is that the number of cases in the infant and adult group remained low at 6.7% and 8.9%, respectively. Within the predominant genotype, E30, E6, and CVA9 represented a surprising genetic diversity with full-length sequences like those obtained from Europe and Asia. This provides a reservoir from which novel strains may potentially emerge and perhaps as an outbreak event. Although the E30, E6, and CVA9 strains detected in this study belonged to a single genogroup within each type, they clustered closely as shown by the phylogenetic analysis.

The high mutation rate and the RNA recombination are responsible for the genetic diversity of NPEV. The recombination increases viral pathogenicity, removes lethal mutations, and increases viral fitness. Of the four NPEV species, *Enterovirus* exhibited the highest rates of recombination particularly between members of the same species. The continuous diversity of the VP1 coding region, which is involved in virus–cell interactions and antibody neutralization, might explain the high endemicity and infection of NPEV [32].

## 5. Conclusions

In conclusion, this study documents for the first time the WGS sequences of E6, CVA9, E30, and EV-A71 circulating in parts of the Russian Federation. The public health impact of enteroviruses has been highlighted by outbreaks of severe neurological disease that mimicked polio but was linked to non-polio enteroviruses. Considering this, we hypothesize that NPEVs may predominate in their ability to cause paralysis after polio is completely eradicated. Thus, investigating non-polio enteroviruses linked to neurological effects becomes essential.

## Figures and Tables

**Table 1:** Patients and sample types.

	Infant Group 0 to 2 y.o.	Preschool Children Group 3 to 6 y.o.	Schoolchildren Group 7 to 17 y.o.	Adults 18+ y.o.	Total
Stool specimens	3	15	26	4	48
Pharyngeal swabs	6	18	32	2	58
CSF	6	28	71	14	119
Total	15 (6.7%)	61 (27.1%)	129 (57.3%)	20 (8.9%)	225

CSF: cerebrospinal fluid, y.o.: years old.

**Table 2:** Species and types of NPEV isolated in aseptic meningitis cases in different subjects of the Ural Federal District and Western Siberia, *n* (%).

Species/Types *	Sverdlovsk Oblast	Chelyabinsk Oblast	Tyumen Oblast	Kurgansk Oblast	Khanty-Mansi Autonomous Okrug	Omsk Oblast	Tomsk Oblast	Novosibirsk Oblast	Total
Coxsackievirus A2	4 (2.5)	0	0	0	0	0	0	0	4 (2.5)
Coxsackievirus A4	2 (1.2)	0	0	0	0	0	0	0	2 (1.2)
Coxsackievirus A5	3 (1.9)	0	0	0	0	0	0	0	3 (1.9)
Coxsackievirus A6	3 (1.9)	0	0	0	1 (0.6)	0	0	0	4 (2.5)
Coxsackievirus A10	1 (0.62)	0	0	0	0	0	0	0	1 (0.62)
Enterovirus A71	1 (0.62)	0	0	0	0	0	0	0	1 (0.62)
***Enterovirus alphacoxsackie #***	**14 (8.8)**	**0**	**0**	**0**	**1 (0.6)**	**0**	**0**	**0**	**15 (9.4)**
Coxsackievirus A9	17 (10.7)	0	0	0	4 (2.5)	2 (1.2)	2 (1.2)	0	25 (15.6)
Coxsackievirus B2	1 (0.62)	3 (1.9)	0	0	0	1 (0.62)	0	0	5 (3.1)
Coxsackievirus B3	0	1 (0.62)	0	0	0	0	0	0	1 (0.62)
Coxsackievirus B4	0	0	0	0	1 (0.62)	0	0	1 (0.62)	2 (1.2)
Coxsackievirus B5	1 (0.62)	1 (0.62)	0	2 (1.2)	5 (3.1)	0	0	1 (0.62)	10 (6.2)
Echovirus E6	8 (5)	0	0	0	10 (6.2)	1 (0.62)	0	12 (7.5)	31 (19,4)
Echovirus E7	1 (0.62)	0	0	0	0	0	0	0	1 (0.6)
Echovirus E9	3 (1.9)	0	0	0	4 (2.5)	1 (0.62)	0	0	8 (5)
Echovirus E11	1 (0.62)	0	1 (0.62)	0	0	1 (0.62)	0	1 (0.62)	4 (2.5)
Echovirus E13	0	0	0	0	0	0	0	1 (0.62)	1 (0.62)
Echovirus E14	0	0	0	0	4 (2.5)	0	0	0	4 (2.5)
Echovirus E19	0	0	0	0	0	0	0	4 (2.5)	4 (2.5)
Echovirus E25	0	0	0	3 (1.9)	2 (1.2)	0	0	4 (2.5)	9 (5.6)
Echovirus E30	11 (6.9)	0	0	3 (1.9)	25 (15.6)	0	0	0	39 (24.4)
***Enterovirus betacoxsackie #***	**43 (26.9)**	**5 (3.1)**	**1 (0.62)**	**8 (5)**	**55 (34.4)**	**6 (3.8)**	**2 (1.2)**	**24 (15)**	**144 (90)**
**Rhinovirus B**	**0**	**0**	**1 (0.62)**	**0**	**0**	**0**	**0**	**0**	**1 (0.62)**
**Total**	**57 (36)**	**5 (3)**	**2 (1)**	**8 (5)**	**56 (35)**	**6 (4)**	**2 (1)**	**24 (15)**	**160**

* Species are represented in bold. # The values in the species rows (in bold) are a subtotal of the type data.

**Table 3:** Demographic data and clinical characteristics of NGS sequences of NPEV strains.

Sample No.	Accession No.	NPEV Genotype	Sample Type	Year of Isolation	Age (Years)	City, Federal Subject	Genome Size (bp)
1086_22	PP236447.1	EV-A71	Nasopharyngeal	2022	5	Ekaterinburg, SO	7283
2831_22	PV822004.1	CVB5	Feces	2022	7	Ekaterinburg, SO	7236
2827_22	PV822003.1	E11	Feces	2022	13	Ekaterinburg, SO	7430
2826_22	PV822002.1	E6	Feces	2022	8	Ekaterinburg, SO	7381
2819_22	PV822001.1	E6	CSF	2022	4	Ekaterinburg, SO	7378
2808_22	PV822000.1	E6	Feces	2022	7	Ekaterinburg, SO	7410
2806_22	PP236450.1	E6	Feces	2022	3	Ekaterinburg, SO	6616
787_22	PP236449.1	E6	CSF	2022	5	Omsk, OO	5820
2813_22	PP236448.1	E6	Feces	2022	57	Ekaterinburg, SO	7482
1173_23	PV821999.1	E30	CSF	2023	12	Kurgan, KO	7266
832_23	PV821997.1	E30	Nasopharyngeal	2023	15	Surgut, KMAO	7414
2816_22	PP236452.1	CVA9	Feces	2022	7	Ekaterinburg, SO	7650
2805_22	PP236451.1	CVA9	Nasopharyngeal	2022	24	Ekaterinburg, SO	7410
1188_23	PQ572232.1	EV-B87	CSF	2023	7	Surgut, KMAO	6455
1326_23	PQ572231.1	EV-B80	CSF	2023	6	Ekaterinburg, SO	6943
1117_23	PQ572230.1	EV-B80	CSF	2023	12	Kurgan, KO	6858

Abbreviations: CSF = cerebrospinal fluid, SO = Sverdlovsk oblast, KO = Kurgansk oblast, KMAO = Khanty-Mansi autonomous okrug, bp = base pair.

## Data Availability

The original data presented in the study are openly available in GenBank (https://www.ncbi.nlm.nih.gov/genbank/, accessed on 14 December 2025) at PP596632–PP596783; OQ696786–OQ696793; PV821997; PV821999–PV822004; PP236447–PP236452; PQ572230–PQ572232.
